# Improved hole injection for blue phosphorescent organic light-emitting diodes using solution deposited tin oxide nano-particles decorated ITO anodes

**DOI:** 10.1038/s41598-019-39451-4

**Published:** 2019-02-20

**Authors:** Seung Il Lee, Geum Jae Yun, Jin Wook Kim, Gregory Hanta, Kunyu Liang, Lazar Kojvic, Lok Shu Hui, Ayse Turak, Woo Young Kim

**Affiliations:** 1 Department of Electronic Display Engineering, Hoseo University, Asan, 31499 South Korea; 2 Department of Engineering Physics, McMaster University, Hamilton, Ontario, L8S 4L7 Canada; 3 Department of Electrical and Electronic Engineering, The University of Hong Kong, Pokfulam Road, Hong Kong, China

## Abstract

Blue phosphorescent organic light-emitting diodes (PHOLEDs) were fabricated with tin oxide (SnO_x_) nano-particles (NPs) deposited at the ITO anode to improve their electrical and optical performances. SnO_x_ NPs helped ITO to increase the work function enhancing hole injection capability. Charge balance of the device was achieved using p- and n-type mixed host materials in emissive layer and the devices’ luminance and maximum external quantum efficiency (EQE) increased about nearly 30%. Tuning the work function using solution processed NPs allows rapid optimization of device efficiency.

## Introduction

The evolution of organic light-emitting diodes (OLED) into flat panel display and solid state planar lightning applications has been the result of much development in luminous efficiency, low power consumption and life-time. Among the most significant was the development of doped guest-host matrix systems that utilizing both singlet and triplet excitons for emissions^1–4^. Such phosphorescent OLEDs (PHOLEDs) have significantly higher luminous efficiencies than their fluorescent counterparts, due to the potential for 100% internal quantum efficiency^2^. Further improvements in device performance, such as higher brightness and efficiency, can occur by promoting exciton recombination probability with improved charge injection at interfaces and balance of charge carriers in the emission layer to maximize the formation of photons without losses^5^.

In blue PHOLEDs, the blue dopant has a large highest occupied molecular orbital (HOMO) - lowest unoccupied molecular orbital (LUMO) energy band gap, in order to generate blue emission. As a result, the host material should also have a larger energy band gap as well as higher triplet energy level than the blue dopant material allowing Dexter energy transfer and confinement of the triplet excitons. Furthermore, the hole transport material should have high triplet energy level to prevent triplet excitons diffusion into the hole transport layer (HTL) as triplet excitons have longer life-time before they generate phosphorescence and electron mobility is typically faster than hole mobility as voltage increases.

Common materials with large triplet energies used for blue PhOLEDs include, blue dopant material bis[3,5-difluoro-2-(2-pyridyl)phenyl-(2-carboxypyridyl)]-iridium (FIrpic) (T_1_ = 2.7 eV), host for blue phosphorescent dyes, 1,3-Bis(*N*-carbazolyl)benzene (mCP) with a large HOMO-LUMO energy gap as well as high triplet energy (=2.9 eV) and HTL materials 1-Bis[4-[N,N-di(4-tolyl)amino]phenyl]-cyclohexane (TAPC) T_1_ = 2.9 eV and a very deep HOMO level (5.9 eV)^6^. In bottom emission PHOLEDs, generally indium tin oxide (ITO) is used for transparent conducting anodes (ϕ = 4.7 eV), It is generally difficult to inject holes directly into mCP from ITO anodes or a typical HTL such as TAPC due to the large hole injection barrier at the interface. As a result of the large hole injection barriers at the ITO/HTL interface, improvement of carrier injection in blue PHOLEDs is expected to have a more significant effect on the device performance than for green or red PHOLEDs and OLED structures.

A variety of approaches have been used to regulate the work function of ITO including plasma treatment^7^, passivation with surface-active species^8^, chemical and physical treatments^7,8^, halogenation^9^, and deposition of high-work- function metals, insulating materials, and oxides^9–12^. Particularly, inserting metal oxide thin film layers such as MoO_3_, WO_3_, and V_2_O_5_ between ITO and HTL by thermal evaporation was seen to reduce the energy barrier between ITO and HTL, enhancing hole injection at the interface in OLEDs^13^. As ITO has a very heterogenous surface, the thickness of these buffer layers are critical to optimizing device performance and sub-monolayer films are highly effective in tuning device performance^12,14^. As thermal evaporation can be difficult to control in the sub-monolayer deposition regime for many interlayer materials, a solution based approach can allow more control over the device properties^10,15^. The possibility of tailoring the work function to match the energy level of the active organic layer is of great interest in the fabrication of organic devices to form barrier-free Ohmic contacts.

Tin dioxide (SnO_2_) is an n-type metal oxide semiconductor widely used for gas sensors^16,17^, ion battery electrodes^18^, electrodes for electrochromic devices^19^, and interlayers in inverted organic solar cells^20^. Because of its transparency, work function of nearly 4.8 eV at bulk state^21^, and tunable electrical conductivity with oxygen vacancy concentration and size^22,23^, it is also a potentially interesting interlayer for OLEDs.

Due to their n-type character, SnO_2_ has been successfully used as an electron injection layer in inverted OLEDs^24^ and organic photovoltaics (OPVs)^20,25–27^. For instance, solution based SnO_2_ NPs were deposited as an electron injection layer (EIL) in an inverted OLED structure to eliminate the energy barrier between the metal anode and electron transport layer (ETL)^24^. However, it has not been widely used as a hole injection layer.

Our interest in using it here stems from three major factors. First, it has been shown that electron acceptors can effectively tune the hole injection properties for regular device architectures^14,28,29^. Second, low temperature growth of tin oxides often results in mixed phases, with SnO_2_ and SnO coexisting as a result of the metastable nature of SnO^30,31^. Mixed phase alloying has been an effective method of tuning the electronic properties of SnO_x_ NPs^32^. Finally, the surface of ITO is known to be tin-rich, with SnO_2_-like crystalline domains^33–35^. These SnO_x_ domains are generally thought to be sites of the highest electrical activity^34^. The ITO work function can be controlled by manipulating the proportion of Sn^4+^ ions at the surface with sputtering^36^. By deliberately introducing Sn^4+^ components with submonolayer coverage of SnO_x_ NPs, we propose an alternative method of controlling the surface electronic structure of ITO for better matching with the deep HOMO of novel HTLs.

In this paper, we introduced SnO_x_ NPs dispersed on ITO anodes using reverse micelles as a tunable transparent metal oxide material for improving bottom emission blue PHOLEDs electrical and optical properties by improving hole injection at the organic/inorganic interface. By using a solution approach, a uniform distribution of NPs can be deposited on the ITO surface, allowing control and flexibility in the device design.

Increasing the injection of holes using the interfacial NPs layer requires the introduction of a graded mixed-host emitting layer structure to optimize charge balance and improve the EQE by about 30%.

## Results

**Figure 1:**
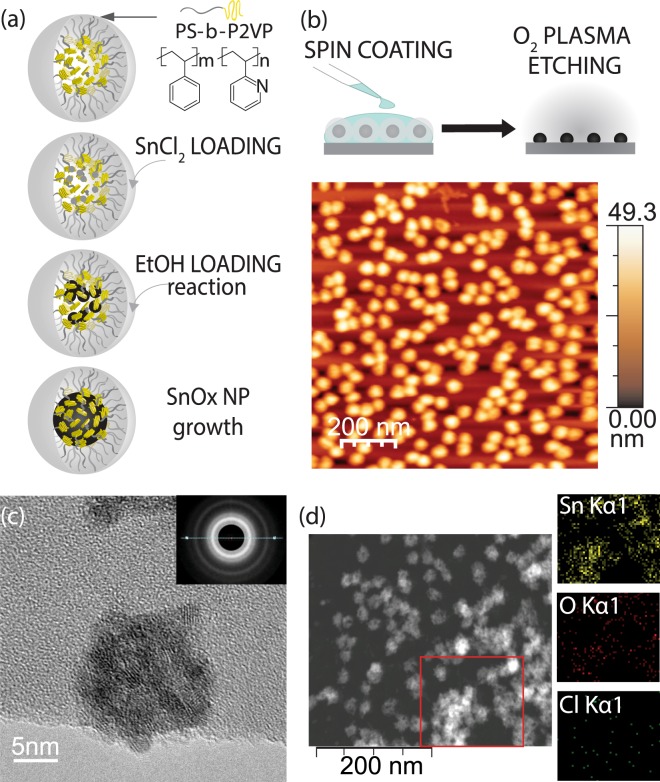
(**a**) Schematic of formation mechanism of SnO_x_ NPs using a PS-P2VP diblock co-polymer reverse micelle. Sequential loading of the precursors salt results in the formation of polycrystalline NPs from the amalgamation of nucleated nanocrystalline domains. (**b**) Atomic force micrograph of monolayer arrays of NPs formed by spin coating and O_2_ plasma etching. (**c**) High resolution transmission electron micrograph of polycrystalline SnO_x_ NPs. Inset shows the selective area electron diffraction pattern. (**d**) Energy dispersive electron spectroscopy elemental maps of NPs deposited on a TEM grid showing the distribution of Sn, O and Cl.

Following the reverse micelle deposition (RMD) process shown schematically in Fig. 1(a) results in a uniform sub-monolayer array of SnO_x_ NPs (Fig. 1(b)). By limiting the reaction region within the micelles, a mean size of 31.6 ± 2.6 nm, with polydispersity indices^37^ of 0.08, were achieved (see Supplemental S1). These particles are large enough to form a sub-monolayer distribution on the ITO surface, while still retaining the electronic properties of bulk tin oxides. As the ITO surface roughness is similar to the mean particle size (RMS = 26.7 nm), the NPs do not increase the roughness. With submonolayer coverage, there appears to be a slight smoothening effect of adding NPs, but the overall surface is substantially similar (see Supplemental S2). To confirm the reproducibility of these results, multiple batches of particles with the same loading ratios were produced on both Si and ITO.

As shown in the EDXS elemental maps of the NPs (Fig. 1(d)) and XPS analysis (Supplemental Fig. S-5), the addition of ethanol to the SnCl_2_ loaded micelles almost completely consumes the chloride, yielding polycrystalline SnO_x_ NPs (Fig. 1(c)). Selective area diffraction (inset of Fig. 1(c)) yields powder rings consistent with the 110, 101, 211 and 112 planes of rutile SnO_2_^38,39^ (see Supplemental S3). XPS characterization (see Supplemental S4) indicates that the tin chloride precursor, solvent and polymeric micelles are completely removed during the multi-step formation, as expected. High resolution core level analysis shows spectra consistent with SnO_2_ NPs^40^, having a Sn 3d_5/2_ core level peak at 437.35 eV. This is offset by 1.1 eV from bulk SnO_2_^41,42^ due to a size dependent shift, also observed for LiF NPs produced by the reverse micelle method^9^. The calculated Sn:O atomic weights suggests an oxygen deficient sub-stoichiometric compound is formed. As oxygen vacancies are known to intrinsically *n*-dope SnO_2_, a deficient structure is beneficial in maintaining high conductivities in the NPs dispersed surface. This could also be due to the coexistence of the thermodynamically stable rutile phase with metastable SnO, though it is difficult to confirm due to the similarity in the Sn ^2+^ and ^4+^ 3d core level positions^41,43,44^.

**Figure 2:**
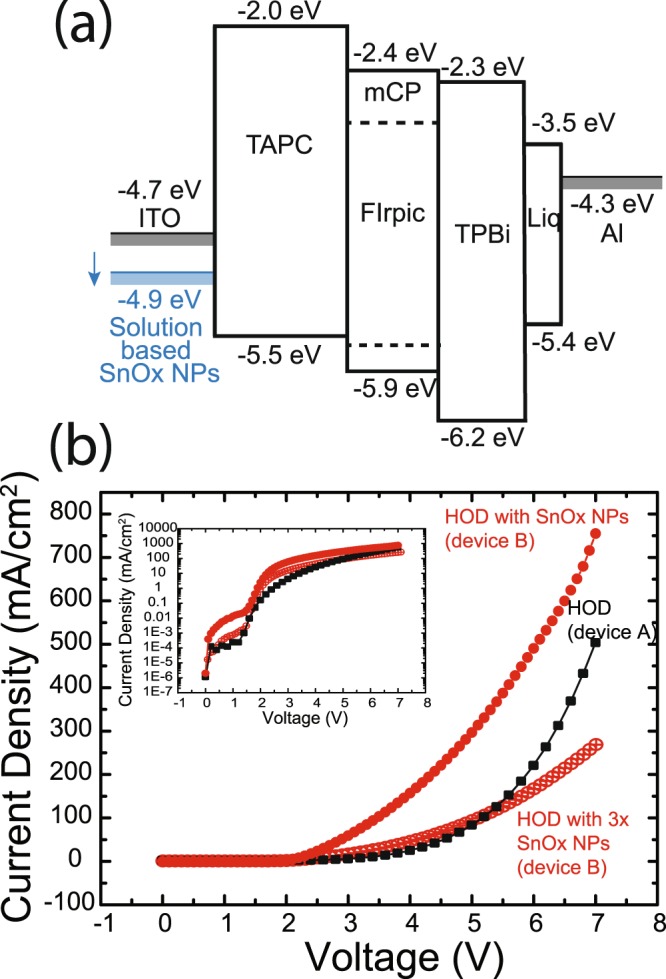
(**a**) Schematic band diagram of PHOLED devices showing the hole injection lowering effect of the SnO_x_ NPs. (**b**) Current-density voltage (J–V) curves for hole only devices based on TAPC with and without SnO_x_ NPs (device A and B). Submonolayer NP and complete films (3x) are shown for SnO_x_ NPs. All devices were exposed to 30 min of O_2_ plasma etching.

Figure 2(a) shows the expected band structure of our blue PHOLED device. The introduction of SnO_x_ NPs increases the work function of the ITO by 0.27 eV as determined by photoemission yield spectroscopy (see Supplemental S5), similar to previous work with RMD sol-LiF^12^. Though SnO_2_ is generally thought of as an n-type semiconductor, and therefore has been recently introduced into inverted type structures as an electron injecting layer^20,24–26^, this increase in surface work function with electron accepting organic molecules has been observed previously^14,28,29^. Hong *et al*. saw a similar increase in ITO work function with a thin layer of C_60_ at the interface between ITO and NPB^29^. It may also be due to the increase in the Sn^4+^ ions on the surface. ITO is known to have a Sn-rich surface^33^, with the work function controlled by the relative abundance of Sn^4+^ ions on the surface. Che *et al*. showed that decreasing the Sn^4+^ fraction with Ar^+^ sputtering led to a decrease in the surface work function^36^. The high work function of ITO generally after cleaning was established to be the result of fully oxidized SnO_2_ domains at the Sn-rich surface of ITO^36^. As the ITO surface structure is generally inhomogenous, with submonolayer coverage of SnO_x_ NPs, we are likely deliberately increasing the Sn^4+^ fraction on the surface, leading to an increased work function, relative to the exposed ITO areas. This shift therefore would be due to the intrinsically higher work function of SnO_x_ as compared to ITO (~0.1–0.2 eV)^21^. The density of dipoles is increased at the surface by isolated SnO_x_ NPs and their interactions decrease the effect of the surface dipole moment^12,15,45,46^.

As this moves the anode work function closer to the HOMO energy level of the TAPC as HTL, it lowers the barrier to hole injection at the ITO/HTL interface. This adjustment of the surface work function has been used to improve performance in both OLEDs and OPVs using a variety of dipole modifying approaches including plasma cleaning, chemical modification and presence of interlayers. Both Schottky thermionic emission and Folwer-Nordheim tunneling (see Supplemental Equations S1 and S2) will be improved by the reduction of the barrier, and will improve as a function of voltage.

Another possibility is the simultaneous coexistence of SnO_2_ with SnO or carbon incorporation into the NPs. Figure 1(d) shows that the NPs tend to be a polycrystalline agglomerate of smaller particles with amorphous regions that could be C, or amorphous Sn oxides. Carbon is known to stabilize NPs and alloying with C has been an effective method of tuning the electronic properties of SnO_x_ NPs^32^. Given our use of diblock co-polymer micelles for the formation of the NPs, it is likely that some carbon has been incorporated into the SnO_x_ matrix.

Alternatively, there could be amorphous and/or Sn^2+^ phases incorporated into the NPs. SnO has been used for high mobility p-type thin film transistors^47–49^, due to its high hole conductivity through divalent tin vacancies. Though the SAD data is not consistent with stannous tin monoxide, SnO_2_ and SnO are known to coexist during low temperature deposition as a result of the metastable nature of SnO^30,31^. Due to the similarity of the Sn 3d core level positions for both Sn ^2+^ and Sn^4+^ ions^41,43,44^, it is difficult to confirm the existence of SnO. The presence of p-type SnO, possibly in the amorphous portions, could improve the injection of charge carriers.

The phenomenon of greater hole injection can be observed in HOD tests based on ITO/TAPC (100 nm)/Al (120 nm). To ensure that the effect is not due to the plasma etching of the ITO surface, the device without NPs was also plasma etched for 30 mins before deposition of TAPC. As shown in the J–V curves of Fig. 2(b), with the addition of SnO_x_ NPs, higher current density is detected at low voltage and continues to increase as the voltage increases, resulting in a higher slope. Consequently, HODs with SnO_x_ NPs obtained nearly 450 mA/cm^2^ of current density but reached only at 193 mA/cm^2^ without SnO_x_ NPs at 6V, with higher charge carrier effect in the space charge limited current (SCLC) curve, as expected. However, the improvement in injection occurs only when there is a submonolayer coverage of NPs on the ITO surface. As seen in Fig. 2(b), depositing multiple layers of NPs until there is a closed layer (3x spin coating and plasma etching) results in a loss of hole injection.

Recently Mazhari, Rivizi and Mantri^50,51^ proposed a new metric, $G=\frac{dlog(J)}{dlog(V)},$ to determine the onset of various current regimes of organic diodes based on the derivative of current density with respect to voltage. As derivative functions are more sensitive to slope changes, such a metric as a function of voltage shows a maximum at the transition voltage between an exponential current based on overcoming the potential barrier at the electrode, and the power law dependent trap-limited and SCLC^51^. It is possible to observe more than one maximum, particularly in systems with traps, suggesting multiple conduction regimes as a function of applied voltage. In a unipolar device, this transition voltage should be proportional to the built-in potential, driven by the difference between the work function of the electrodes^50^. Therefore, changes to the transition voltage can indicate changes to the barrier for charge flow through the device.

**Figure 3:**
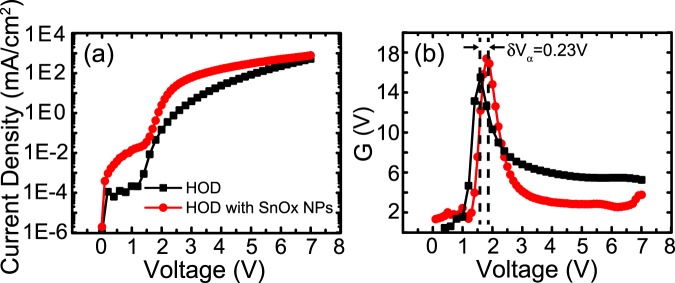
(**a**) Current-density voltage (J–V) curves for hole only devices based on TAPC with and without SnO_x_ NPs (device A and B). Both devices were exposed to 30 min of O_2_ plasma etching. (**b**) G–V characteristics where G represents the derivative function, $G=\frac{dlog(J)}{dlog(V)}$. The peak in G(V) represents the transition voltage between exponential and power-law dependent current-voltage regimes.

Figure 3 shows the J–V and G–V curves for the HODs, where an increase in the transition voltage of 0.23 V is observed due to the introduction of NPs. This is consistent with the expected barrier lowering observed with the increase in the ITO surface work function. Additionally, beyond the transition voltage, G(V) is proportional to the power law exponent, *m*, of the J–V characteristics, and can indicate if the system has entered into a trap-limited (m > 2) or space-charge limited conduction regime (m ≈ 2)^50^. Without SnO_x_ NPs, the exponent is over 4 (4.3 ± 0.1 from 8 devices), suggesting that the transition voltage indicates the onset of trap-limited current. With SnO_x_ NPs, the exponent drops to 1.68, indicating that the increased injection of holes allows the device to reach the SCLC regime under lower applied voltage.

**Figure 4:**
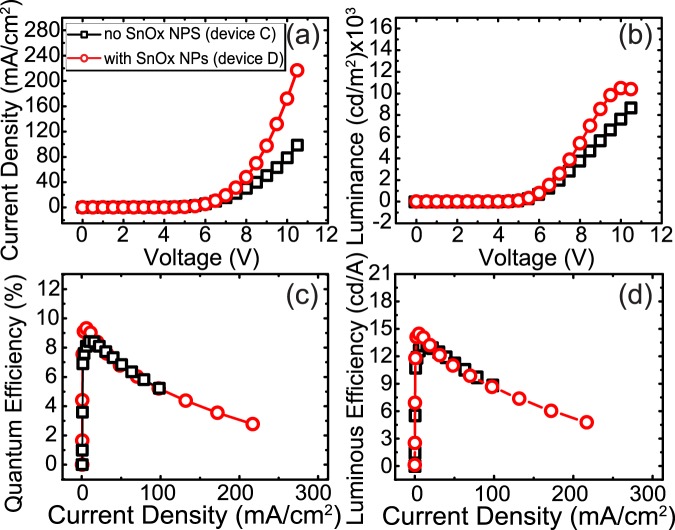
Electric characteristic of blue PHOLEDs with p-host EML, including SnO_x_ NPs (**a**) current density (J–V) (**b**) luminance (L–V) (**c**) quantum efficiency and (**d**) luminous efficiency as a function of current density.

When a single submonolayer layer of SnO_x_ NPs were included in blue PHOLEDs using a p-type host material (mCP) for the emitting layer, devices with a NPs interlayer showed higher luminance and current density as shown in Fig. 4 and Table 1, as was expected from the HOD tests. The luminous and quantum efficiencies were also increased by roughly 10–11% by using the SnO_x_ NPs as shown in Fig. 4(c,d) compared to the device without NPs (device C). To get adequate statistics, we examined a number of devices and reported the average values, along with the error in Table 1. Devices with SnO_x_ NPs appear more consistent, with a slightly narrower distribution of efficiency values compared to devices without ITO surface modification. This might arise from the slight smoothening of the ITO surface with NPs or introducing a homogenous distribution of Sn^4+^ containing NPs on the typically inhomogenous ITO surface. Decoration of inhomogenous ITO with LiF NPs has been previously shown to produce more uniform potential surfaces^9^. From our device results, it appears that at lower voltages, the current density is bulk diffusion limited rather than injection limited, with more holes swept into the EML, and the overall yield of excitons is increased. However, considering the increased number of holes in the EML observed in device D, we expected a greater improvement in the luminous and quantum efficiencies. A mismatch in the current density and efficiency suggests a charge imbalance within the device^52–54^. In such devices, improving the injection of charge carriers that are already in excess may improve the absolute current density and luminance due to the greater probability of charge recombination, but lead to minimal increases or even lowered efficiency if the number of excess holes, ${N^{\prime\prime} }_{h}$, greatly exceeds the number of electron hole pairs $({N^{\prime} }_{e}={N^{\prime} }_{h})$. If the hole and injection currents are not equal, the charge balance factor, γ (Eq. 1), may be less than 1, limiting the quantum efficiency.

1 $${\rm{\gamma }}=\frac{{J^{\prime} }_{Cn}-{J^{\prime} }_{An}}{J}=\frac{{J^{\prime} }_{Cp}-{J^{\prime} }_{Ap}}{J}=\frac{{N^{\prime} }_{h}+{N^{\prime} }_{e}}{{N^{\prime\prime} }_{h}+{N^{\prime} }_{h}+{N^{\prime} }_{e}}$$

**Table 1:** Average device efficiency values for blue PHOLED devices.

Device	EQE(MAX)	EQE(at6V)	LE(MAX)	LE(at6V)	PE(MAX)	PE(at6V)
p-host (device C)	8.9 ± 0.4	8.5 ± 0.6	14.2 ± 0.7	13.5 ± 1.0	7.5 ± 0.5	7.0 ± 0.5
p-host/SnO_x_ NPs (device D)	9.2 ± 0.2	9.2 ± 0.2	14.5 ± 0.4	14.5 ± 0.4	7.9 ± 0.3	7.6 ± 0.2
mixed host (device E)	8.6 ± 0.8	8.4 ± 0.7	13.9 ± 1.2	13.5 ± 1.0	8.0 ± 0.4	7.1 ± 0.5
mixed host/SnOx NPs (device F)	11.0 ± 0.3	10.5 ± 0.2	17.8 ± 0.5	17.3 ± 0.3	10.4 ± 0.7	9.0 ± 0.2

Generally, transporting holes is relatively easier than electrons in most organic devices. TAPC as HTL and mCP^55^ as the EML host material have hole mobilities of ~1.0 × 10^−2^ and 3.2 × 10^−4^ cm^2^/Vs respectively, orders of magnitude larger than for the ETL layer, TPBi, which has a typical electron mobility of ~3.3–8 × 10^−6^ cm^2^/Vs^56,57^. In our PHOLED structure, we used mCP as a host material because of the high triplet energy and high quantum yield when used with FIrpic as a blue dopant^6^. Although mCP has only a slightly lower electron mobility than hole mobility (*μ*_*e*_ = 2.0 × 10^−4^ cm^2^/Vs)^55^, the mismatch in the charge carriers leads to a buildup of excitons forming at the mCP/TPBi interface and diffusing into the EML. This suggests that as more holes are injected with the barrier lowering effect of the SnO_x_ NPs, the number of excess holes in the EML increases, increasing current density while keeping the luminous efficiency almost unchanged.

**Figure 5:**
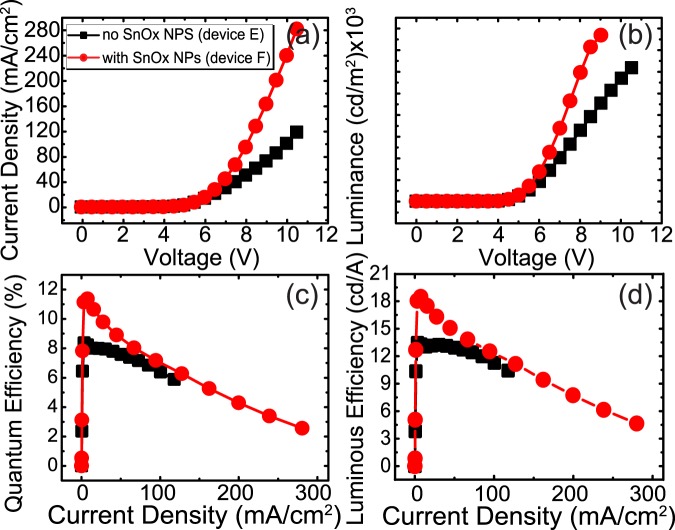
Electric characteristic of blue PHOLEDs, including SnO_x_ NPs, with a p-/n- mixed host EML layer for charge balance optimization (**a**) current density (J–V) (**b**) luminance (L–V) (**c**) quantum efficiency and (**d**) luminous efficiency as a function of current density.

Therefore, to improve performance, we optimized the charge balance in the device, by incorporating a graded p-/n-type mixed host for part of the emission layer^58,59^. Using mCP:TPBi with a 1:2 ratio at the right side of the EML significantly improved the electron mobility, as has also been observed in white PHOLEDs^54^. TPBi is a well known n-type electron transporting material and has also been used as an n-type host material^59,60^. In such a structure without NPs (device E), due to rapid electron injection into p-/n- mixed host, the current density and luminance is again improved, but the quantum efficiency is almost unchanged, this time due to the increased density of electrons. The increased electron injection led to a decreased efficiency roll-off due to the extension of the recombination zone compared with the standard device with only a p-type host (device C). Additionally, modifying the EML host results in an approximately 0.75 V decrease in the turn-on voltage, but only a slight change in the x,y chromaticity coordinates (see Supplemental Fig. S-6). Having both increased hole injection with a SnO_x_ NPs, and increased electron mobility with a p-/n-type mixed host achieving higher charge balance, the charge optimized structure results in ~30% increase in the quantum efficiency comparing device E and F (Fig. 5 and Table 1).

This is a much larger increase than that observed for more traditional HIL, such as thin films of MoO_3_, WO_3_, and Vd_2_O_5_ in blue PHOLEDs. Chiu and Chuang^13^ produced blue PHOLEDs with various thin oxide interlayers deposited by sputtering. Though they proposed a similar mechanism of reducing the barrier at the interface by using high work function interlayers, they actually observed no change or a loss in the absolute current density or luminance for every interlayer. Additionally, unlike in our system where we observed ~30% improvement with an optimized device for the EQE, power efficiency and luminance efficiency, they only observed improvements varying from 2–18%, with average increase in the EQE of 3%, PE of 11% and current efficiency of 7%. This may be a result of not optimizing the EML to accommodate increased hole current, or due to the lack of active Sn^4+^ sites on the surface to improve the charge injection.

## Conclusions

Originally blue PHOLEDs are known to have low luminous efficiency and high driving voltage due to large barriers to charge injection at interfaces. However, we demonstrated highly efficient blue PHOLEDs by tuning the surface electronic structure of the anode using solution processed SnO_x_ NPs on the ITO. The current density was significantly increased in both HODs and in blue PHOLEDs due to the increased hole injection from ITO to the TAPC HTL with a submonolayer coverage of SnO_x_ NPs. Increasing the coverage to achieve a complete layer significantly suppressed hole injection. Charge balance was optimized using a mixed host EML to improve luminance, luminous, quantum and power efficiency. In our study, turn-on voltage was not significantly reduced, suggesting that further optimization is possible with this system. Deliberately introducing Sn^4+^ components with submonolayer coverage of SnO_x_ NPs is a versatile method of controlling the surface electronic structure of ITO. Reducing the energy barrier to improve hole injection capability through dispersed NPs on the anodes could replace organic buffer layers in thermally activated delayed fluorescent (TADF) OLEDs or deep blue OLEDs requiring even greater HOMO-LUMO energy band gap EML materials.

## Method

For SnO_x_ NPs synthesized in solution, polystyrene-block-poly-2-vinylpyridine (PS-b-P2VP) di-block copolymer (polymer source, P1330-S2VP) was dissolved in 5 ml o-xylene and stirred for at least 24 hours. After forming spherical reversed micelles, 10 mg tin-chloride (Alfa Aesar Korea, SnCl_2_ anhydrous) and 0.3 ml of ethanol was added sequentially in the solution, with constant stirring of at least 48 hours after each loading step. The solution was then centrifuged to remove any precipitates. A monolayer of SnO_x_-loaded micelles was deposited on various substrates by spin-coating at 2000 rpm, followed by an O_2_ plasma etching under 950 mTorr, 29.6 W for 25 min to remove the polymeric micelles and solvent. Transmission electron microscopy/scanning transmission electron microscopy (JEOL 2010F TEM/STEM) operating at 200 kV was carried out to acquire the morphological and structural information of the SnO_x_ NPs. In addition, the NPs are characterized using energy dispersive x-ray spectroscopy (EDXS) on the STEM. Silicon nitride membrane window TEM grids (0.05 × 0.05 mm × 10 nm, Norcada) were utilized to facilitate the spin coating and oxygen plasma process, which allowed the direct TEM/STEM observation of the NPs. We spin-coated SnO_x_ NPs on Si wafers to confirm monolayer formation by atomic force microscopy (AFM) before fabricating blue OLED devices. AFM was done in air in tapping mode with a phase locked loop (PLL) dynamic measurement board (Asylum). The non-contact silicon tips were OTESPA (Asylum) with a resonance frequency of 300 kHz, a force constant of 26 N/m and a tip radius of curvature 7 nm. The AFM images were processed with WSxM (NanoTec). Work function of ITO with SnO_x_ NPs was measured by photoemission yield spectroscopy.

ITO coated glasses were cleaned in an ultrasonic bath sequentially with deionized water, acetone, deionized water and isopropyl alcohol, and dried in a N_2_ stream. A monolayer of SnO_x_ NPs was deposited onto the pre-cleaned ITO glass substrates, and cleaned again in a N_2_ stream. Hole only devices (HODs) and blue PHOLED devices were fabricated by thermal evaporation under high vacuum conditions of 5.0 × 10^−7^ Torr. The complete device structure was composed of TAPC as HTL, mCP as host material, FIrpic as blue dopant material, 2,2′,2″-(1,3,5-Benzinetriyl)-tris(1-phenyl-1-H-benzimidazole) (TPBi) as ETL, Lithium 8-quinolinolate (Liq) as EIL. The cathode electrode, aluminum, was also deposited by thermal evaporation. The devices were then encapsulated in an inert N_2_ glovebox using a getter sheet. Specific details of the device structures are outlined in Table 2. The electrical and optical characteristics of blue PHOLED as well as HODs were measured by an I-V-L system.

**Table 2:** Details of device structures.

Device No.	Device Structures (Unit : nm)
Device A	ITO/TAPC (100)/Al (120)
Device B	ITO/SnO_x_ NPs/TAPC (100)/Al (120)
Device C	ITO/TAPC (70)/mCP:FIrpic-8% (30)/TPBi (30)/Liq (2)/Al (120)
Device D	ITO/SnO_x_ NPs/TAPC (70)/mCP:FIrpic-8% (30)/TPBi (30)/Liq (2)/Al (120
Device E	ITO/TAPC (70)/mCP:FIrpic-8% (20)/mCP:TPBi (1:2):FIrpic-8% (10)/TPBi (30)/Liq (2)/Al (120)
Device F	ITO/SnO_x_ NPs/TAPC (70)/mCP:FIrpic-8% (20)/mCP:TPBi (1:2):FIrpic-8% (10)/TPBi (30)/Liq (2)/Al (120)

## Supplementary information

Supporting information
