# Supplementary material for: Improved hole injection for blue phosphorescent organic light-emitting diodes using solution deposited tin oxide nano-particles decorated ITO anodes
Source: Sci Rep. 2019 Feb 20;9:2411. doi: 10.1038/s41598-019-39451-4 (PMC6382941; doi:10.1038/s41598-019-39451-4)
Supplement: Supplementary file 1 — Supporting information [file 41598_2019_39451_MOESM1_ESM.docx]

*Supporting Information for*

**Improved hole injection for blue phosphorescent organic light-emitting diodes using solution deposited tin oxide nano-particles decorated ITO anodes**

**Seung Il Lee^1^, Geum Jae Yun^1^, Jin Wook Kim^3^, Greg Hanta^2^, Kunyu Liang^2^, Lazar Kojvic^2^, Lok Shu Hui ^2^, Ayse Turak^2*^ and Woo Young Kim^1,2*^**

*^1^Department of Electronic Display Engineering, Hoseo University, Asan, 31499, South Korea*

*^2^Department of Engineering Physics, McMaster University, Hamilton, Ontario L8S 4L7, Canada*

*^3^Department of Electrical and Electronic Engineering, The University of Hong Kong, Pokfulam Road, Hong Kong, China*

E-mail: [turaka@mcmaster.ca](mailto:turaka@mcmaster.ca), wykim@hoseo.edu

Phone: +1 905 525-9140 ex 23348

# S1. Mean size and polydispersity index of reverse micelle nano-particles

The particle size distributions were more precisely determined by randomly selecting particles in the AFM images. More than 100 particles were analyzed to obtain a statistical representation of the entire population^1^. Gaussian distribution functions were fitted to eliminate unexpected small and large particles to obtain precise statistical values.


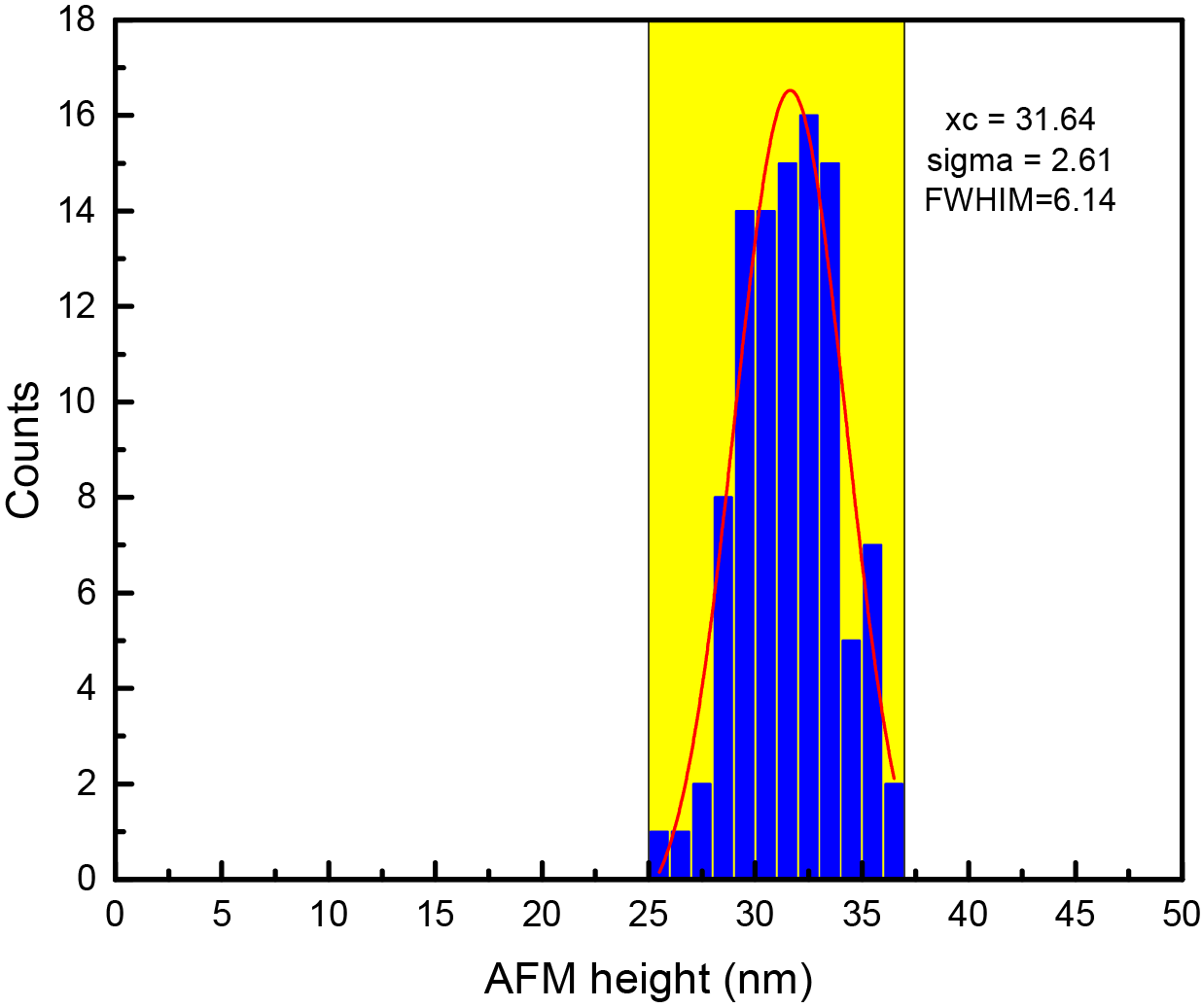


Figure S-1 Height histogram for measured nanoparticles from AFM

# S2. Surface roughness of ITO surfaces coated with reverse micelle nano-particles

#
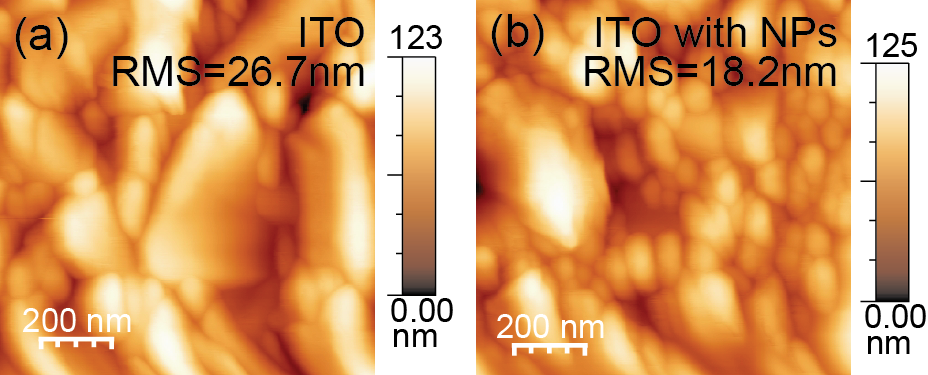


Figure S-2 Atomic force microscopy images for surface roughness determination of (a) bare ITO (b) SnOx nanoparticle decorated ITO

# S3. Crystal structure of reverse micelle nano-particles

Selective area diffraction was performed on nano-particle dispersions spin coated onto silicon nitride membrane window TEM grids (window thickness = 10 nm, window size = 0.05 mmx0.05 mm, Norcada). The extracted d-spacings are consistent with that for the powder diffraction file for rutile SnO_2_, but not for SnO.^2,3^

Table S-1 Measured crystal structure data from SAD region in figure 1

| Peak | 2R (nm^-1^) | d-spacing (nm) | hkl | SnO_2_ JCPDS d-spacing | I/I_o_ | hkl | SnO JCPDS d-spacing | I/I_o_ |
| --- | --- | --- | --- | --- | --- | --- | --- | --- |
| 1 | 5.898 | 0.339 | 110 | 0.3351 | 100 | 112 | 0.3117 | 100 |
| 2 | 7.499 | 0.267 | 101 | 0.2644 | 80 | 020 | 0.286 | 23 |
| 3 | 11.249 | 0.178 | 211 | 0.1765 | 65 | 200 | 0.250 | 22 |
|  |  |  | 200 | 0.2369 | 25 | 220 | 0.188 | 21 |
| 4 | 13.609 | 0.147 | 112 | 0.1498 | 18 | 024 | 0.199 | 14 |


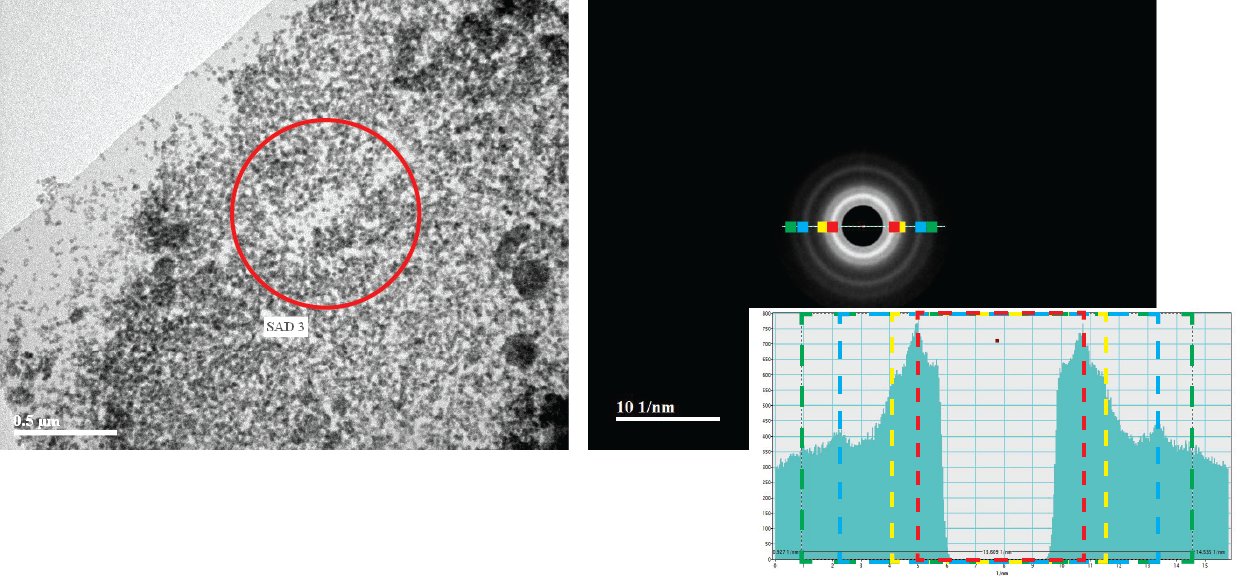


Figure S-3 Selective area diffraction of SnOx nanoparticles on TEM grid within the highlighted red area.

# S4. Chemical composition of reverse micelle nano-particles

The XPS spectra were generated by a monochromated Al Ka source (Thermo K-Alpha) with a photon energy of 1486.6 eV at a pass energy of 100.00 eV and 25.00 eV for wide range survey scans and high resolution individual core level scans. The spot size was 400 mm^2^. The XPS spectra was analyzed with the Thermo Avantage software tool using the Smart background correction option, which is based on the Shirley background correction method. The peaks were then fitted with Gaussian-Lorentzian functions. Samples were Ar sputtered using an Ar cluster source at 8000, 2mm raster for 60s to remove adventitious carbon and oxygen contaminants.


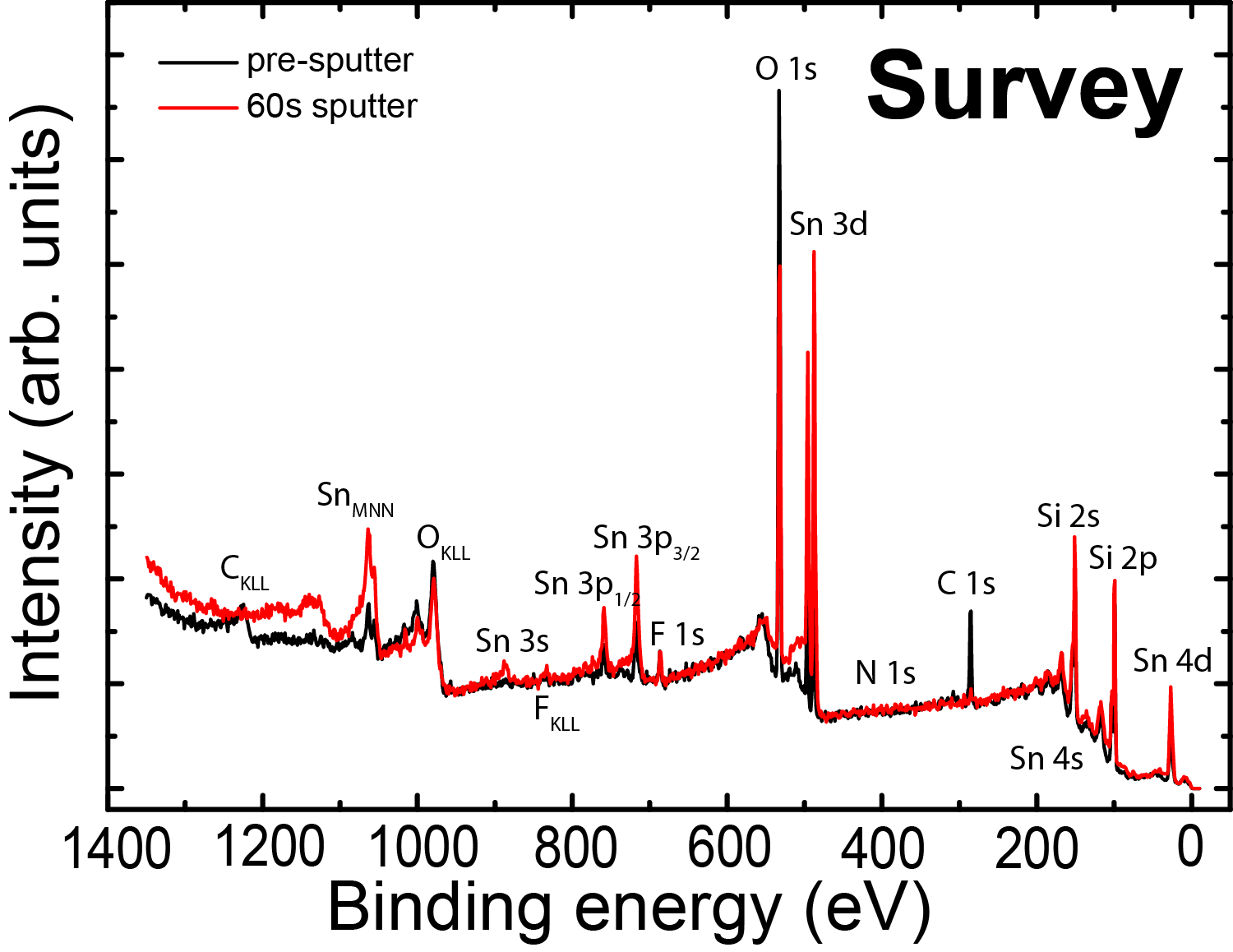


Figure S-4 Wide range survey scan for SnOx nanoparticles before and after 60s Ar sputtering

The wide range survey XPS spectra for the SnOx nanoparticles are shown in Fig. S-3. The major features of Sn are visible for the Sn 4d, 4s, 3d, 3p core levels and MNN Auger peaks^4^, as summarized in table S-2. The spectrum also contains adventitious carbon, nitrogen and oxygen which were mostly removed with Ar plasma etching. Some residual fluorine likely due to the residue from HF etch cleaning of the Si wafer^5^ before deposition is also visible.

Fig. S-4 shows the high resolution core level features. The *Sn 3d* XPS spectra in Fig. S-4 shows a characteristic spin orbit split between the *3d_5/2_* and *3d_3/2_* features, separated by 8.4eV^6^, consistent with SnO_2_. The position of the *3d_5/2_* core level is 1.1eV offset from that of bulk SnO_2_ (~486eV^6–8^). This is likely due to a size dependent core level shift, as this value is consistent with other SnO_2_ nano-particles^9^ and a similar shift has been observed for LiF produced by the reverse micelle method.^10^ Though this higher 3d_5/2_ core level position is consistent with tin chloride (reported at 487.8eV^11^), no clear Cl peak was visible in the nano-particles between 198-199.7 eV as highlighted in Fig S-4; if such a peak exists it appears to be buried within a plasmon peak from the *Si 2s* core level spectra from the substrate.

The *O1s* high resolution core spectra is dominated by adventitious oxygen and SiO_2_ from the substrate, but a small shoulder is visible on the low binding energy side consistent with tin oxides.^7,8^ The calculated stoichiometry from the core level intensities yields 1:1.2 for Sn:O, suggesting a sub-stoichiometric oxide formation. Though the intensity ratio of Sn to O could also suggest a 2+ valence state for Sn, *Sn 3d* electrons in SnO are known to have a lower binding energy than Sn4+^6^ and no second feature was observed in the *Sn 3d* core levels. The other core level features, as well as the electron diffraction results, suggest SnO_2_ formation over SnO. Though a small *N 1s* feature remains, there is no evidence of *C 1s* after Ar sputtering the nano-particle film, indicating that the polymeric micelles have been completely removed during O_2_ plasma etching, as expected.


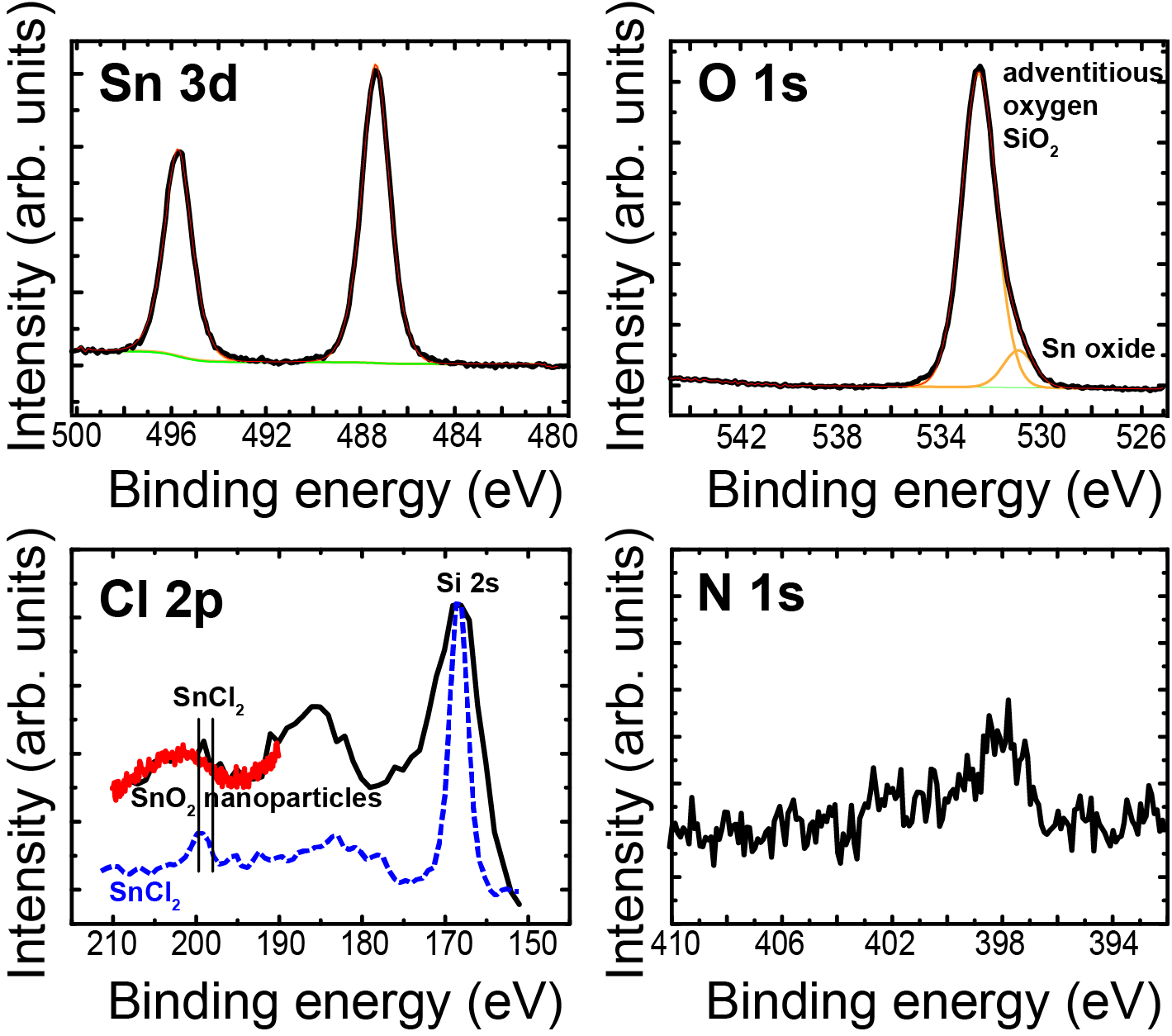


Figure S-5 High resolution core level spectra for SnOx nanoparticles spin coated on a Si wafer. The Sn 3d and O 1s core levels were curve fit to extract the peak centre of mass. The Cl 2p high resolution scan (red) is overlaid with low resolution survey scan for a wider region including the Si 2s. The dotted blue line shows the same region for SnCl_2_ films deposited on Si. The N 1s shows the almost complete removal of the polymeric micelles.

Table S-2 XPS data for survey scans of SnOx nano-particles

| Peak designation | Binding energy (eV) | Visible post Ar sputtering |
| --- | --- | --- |
| Sn related peaks |  |  |
| SnMNN | 1054.27 | Y |
| Sn3s | 880 | Y |
| Sn3p1/2 | 758.26 | Y |
| Sn3p3/2 | 716.17 | Y |
| Sn3d3/2 | 495.75 | Y |
| Sn3d5/2 | 487.35 | Y |
| Sn4s | 133.27 | Y |
| Sn4d | 26.88 | Y |
|  |  |  |
| CKL1 | 1222.27 | N |
| C1s | 284.87 | N |
| N1s | 400.37 | N |
| O1s | 531.84 | Y |
| OKLL2 | 999.27 | Y |
| OKLL1 | 978.27 | Y |
| F1s | 685.86 | Y |
| FKLL | 830.73 | Y |
| Si2s | 150.27 | Y |
| Si2p | 99.27 | Y |

# S5. Work function measurements of nano-particle decorated ITO

The work function, Φ, is the difference in energy between an electron at the Fermi level just inside the surface and at rest in vacuum,^12^ and a key technique to measure it is photoelectron emission yield spectroscopy. When irradiated with a UV source, electrons with a kinetic energy greater than the apparent photoemission threshold, φ, will be ejected from the specimen. The fastest, i.e. most energetic, electrons would be those at the Fermi level, while the slowest electrons would be those with energy equal to Φ. A negative deviation of φ from Φ can be attributed to emission from surface states^13,14^

The work function of the SnOx-modified ITO surface was measured by photoelectron emission yield spectroscopy (PEYS; Riken Keiki AC-2, Tokyo, Japan). A deuterium lamp was used as the UV source, and the incident light was adjusted from hν = 3.4 to 6.2 eV by a grating monochromator. Electrons emitted into air from the solid surface, irradiated by a 4 mm × 4 mm spot, were detected by an air-filled counter (open counter) equipped with two grids—one grid quenched the counter discharge using an external circuit, while the other grid suppressed the positive-ion bombardment. The certified repeatable accuracy for the measurement of the work function using this method is 0.02 eV^15^

As ITO is a degenerate semiconductor, the electronic band structure model for a metal can be applied to analyze the quadratic increase in photoelectron yield with the incident photon energy.^16^ Recognizing that Yield^1/2^ ~ (E_kinetic_ − E_incident_) for E_incident_ N φ, the surface work function Φ can be extrapolated from a fit of the linear portion of the energy curve. The energy curves are presented in Fig. S-5, The bare ITO sample was exposed to the same oxygen plasma treatment conditions as the SnOx covered sample. An increase in work function on the order of 0.13 eV can be expected for oxygen plasma etched ITO^10^ which typically has a work function of 4.7–4.8 eV.


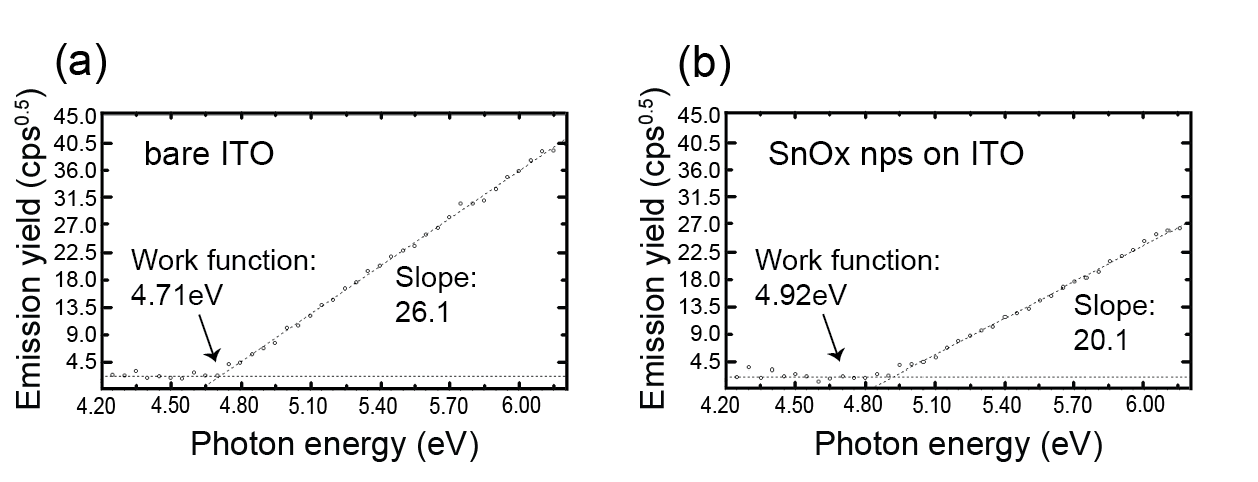


Figure S-6 Photoemission yield spectroscopy for work function determination of (a) bare ITO (b) SnOx nanoparticle decorated ITO.

Two predominate mechanisms exist for the control of injection at the electrode-organic semiconductor interface – Thermionic emission and Fowler-Nordheim tunneling. Both use the tunneling barrier as defined as the difference in the electrode surface work function and the HOMO level of the HTL to control the current density of charge carriers. Decreasing this difference will increase the flux of charge carriers injected into the organic layer.

$J= \frac{4\pi qm^{*}k}{h^{3}}T^{2}\exp\left( -\frac{q\emptyset_{Bn}}{k_{B}T} \right)\left[ \exp\left( \frac{qV}{k_{B}T} \right)-1 \right] (1)$

$J=\left( \frac{q^{3}V^{2}m_{0}}{8\pi h\emptyset_{Bn}m^{*}} \right)\exp\left( -\frac{4\left( 2m^{*} \right)^{0.5}\emptyset_{Bn}^{1.5}}{3hqV} \right) (2)$

# S6. Device details


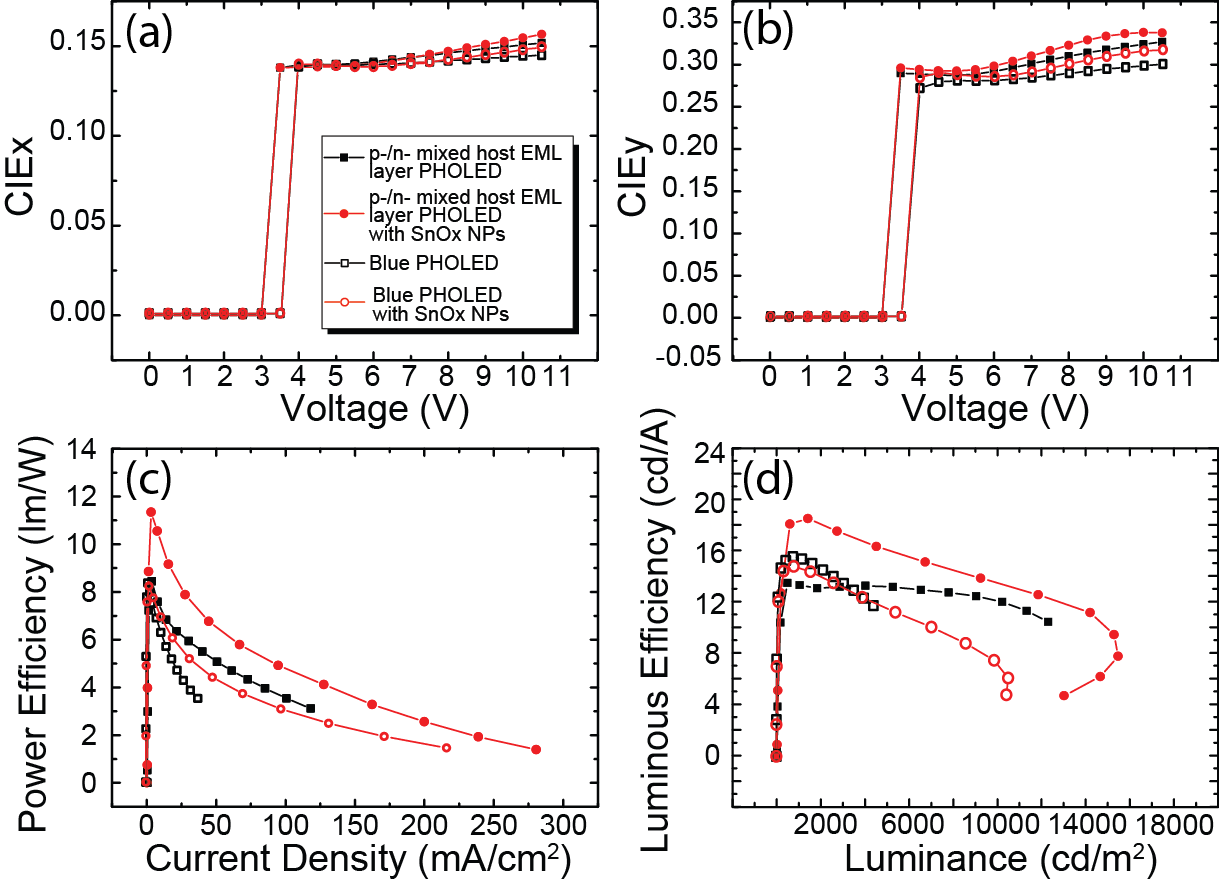
a

Figure S-7 Electric characteristic of blue PHOLEDs, including SnO_x_ NPs (circles); filled corresponds to a p-/n- mixed host EML layer for charge balance optimization (a) x chromaticity (b) y chromaticity (c) power efficiency (d) luminous efficiency as a function of luminance.

References:

(1) Kim, K. Y.; Park, S. B. Preparation and Property Control of Nano-Sized Indium Tin Oxide Particle. *Mater. Chem. Phys.* **2004**, *86* (1), 210–221.

(2) Data, J.--I. C. for D. *Selected Powder Diffraction Data for Metals and Alloys: Data Book*; The Centre, 1978.

(3) Tin Dioxide (SnO2) Crystal Structure, Lattice Parameters, Thermal Expansion. In *Non-Tetrahedrally Bonded Elements and Binary Compounds I*; Madelung, O., Rössler, U., Schulz, M., Eds.; Springer-Verlag: Berlin/Heidelberg, 1998; Vol. 41C, pp 1–2.

(4) Briggs, D. *Handbook X-Ray and Ultraviolet Photoelectron Spectroscopy*; Heyden & Son, Ltd.: London, UK, 1977.

(5) Alam, A. U.; Howlader, M. M. R.; Deen, M. J. Oxygen Plasma and Humidity Dependent Surface Analysis of Silicon, Silicon Dioxide and Glass for Direct Wafer Bonding. *ECS J. Solid State Sci. Technol.* **2013**, *2* (12), P515–P523.

(6) Themlin, J.-M.; Chtaïb, M.; Henrard, L.; Lambin, P.; Darville, J.; Gilles, J.-M. Characterization of Tin Oxides by X-Ray-Photoemission Spectroscopy. *Phys. Rev. B* **1992**, *46* (4), 2460.

(7) Kövér, L.; Kovács, Z.; Sanjinés, R.; Moretti, G.; Cserny, I.; Margaritondo, G.; Pálinkás, J.; Adachi, H. Electronic Structure of Tin Oxides: High‐resolution Study of XPS and Auger Spectra. *Surf. Interface Anal.* **1995**, *23* (7–8), 461–466.

(8) Szuber, J.; Czempik, G.; Larciprete, R.; Koziej, D.; Adamowicz, B. XPS Study of the L-CVD Deposited SnO2 Thin Films Exposed to Oxygen and Hydrogen. *Thin Solid Films* **2001**, *391* (2), 198–203.

(9) Wang, Y. D.; Ma, C. L.; Li, H. D.; Zhang, S. Synthesis and Characterization of the Composite of SnO2 Nanoparticles Coated on SiO2 Microspheres. *Mater. Chem. Phys.* **2008**, *107* (2), 248–253.

(10) Aytun, T.; Turak, A.; Baikie, I.; Halek, G.; Ow-Yang, C. W. Solution-Processed LiF for Work Function Tuning in Electrode Bilayers. *Nano Lett.* **2012**, *12* (1), 39–44.

(11) Alonzo, G.; Bertazzi, N.; Ferraro, J. R.; Furlani, A.; Iucci, G.; Polzonetti, G.; Russo, M. V. Mössbauer, Far-Infrared, and XPS Investigations of SnCl2 and SnCl4 Introduced in Polyconjugated Monosubstituted Acetylene Matrices. *Appl. Spectrosc.* **1995**, *49* (2), 237–240.

(12) Monch, W. On the Physics of Metal-Semiconductor Interfaces. *Rep. Prog. Phys.* **1990**, *53* (3), 221.

(13) Allen, F. G. Work Function, Photoelectric Threshold, and Surface States of Atomically Clean Silicon. *Phys. Rev.* **1962**, *127* (1), 150–158.

(14) Kane, E. O. Theory of Photoelectric Emission from Semiconductors. *Phys. Rev.* **1962**, *127* (1), 131–141.

(15) Jia, J.; Takasaki, A.; Oka, N.; Shigesato, Y. Experimental Observation on the Fermi Level Shift in Polycrystalline Al-Doped ZnO Films. *J. Appl. Phys.* **2012**, *112* (1), 013718.

(16) Sato, Y.; Ashida, T.; Oka, N.; Shigesato, Y. Carrier Density Dependence of Optical Band Gap and Work Function in Sn-Doped In2O3 Films. *Appl. Phys. Express* **2010**, *3* (6), 061101.
